# *Aloe* Polysaccharides Inhibit Influenza A Virus Infection—A Promising Natural Anti-flu Drug

**DOI:** 10.3389/fmicb.2018.02338

**Published:** 2018-09-27

**Authors:** Zhenhong Sun, Cuilian Yu, Wei Wang, Guangfu Yu, Tingting Zhang, Lin Zhang, Jiguo Zhang, Kai Wei

**Affiliations:** ^1^School of Basic Medical Sciences, Taishan Medical University, Tai’an, China; ^2^College of Animal Science and Technology, Shandong Agricultural University, Tai’an, China; ^3^Guangdong Winsun Bio-pharmaceutical Co., Ltd., Guangzhou, China

**Keywords:** *Aloe* polysaccharides, H1N1, antivirus, viral adsorption, transmission electron microscopy

## Abstract

Influenza A virus causes periodic outbreaks and seriously threatens human health. The drug-resistant mutants have shown an epidemic trend because of the abuse of chemical drugs. *Aloe* polysaccharides (APS) extracted from *Aloe vera* leaves have evident effects on the therapy of virus infection. However, the activity of APS in anti-influenza virus has yet to be investigated. Here, we refined polysaccharides from *A. vera* leaf. *In vitro* test revealed that APS could inhibit the replication of a H1N1 subtype influenza virus, and the most obvious inhibitory effect was observed in the viral adsorption period. Transmission electron microscopy indicated that APS directly interacted with influenza virus particles. Experiments on PR8 (H1N1) virus infection in mice demonstrated that APS considerably ameliorated the clinical symptoms and the lung damage of the infected mice, and significantly reduced the virus loads and mortality. Our findings provided a theoretical basis for the development of novel natural anti-influenza agents.

## Introduction

Influenza A virus (IAV) causes acute respiratory distress syndrome, annual epidemics, and occasional pandemics, which have claimed the lives of millions of individuals. Oseltamivir-resistant H1N1 influenza viruses are spread seasonally (Hibino et al., 2017; Leung et al., 2017), and swine-originated H1N1 viruses that have been transmitted to and spread among humans cause outbreaks worldwide (Liu et al., 2017; Ylipalosaari et al., 2017). Highly pathogenic H5N1 avian influenza virus infection continuously threatens poultry and human health (Ly et al., 2017). Some novel recombinant viruses, such as H7N9 and H5NX viruses, have also emerged and affected human health (Pan et al., 2016; Taubenberger and Morens, 2017). These results highlight the limitations of preventive and therapeutic measures against the influenza virus.

Vaccines and antiviral drugs are available for the control of influenza virus infections. Two kinds of antiviral drugs, namely, ion channel and neuraminidase inhibitors, are licensed for use against IAV. Human seasonal influenza vaccines, including H1N1 and H3N2, and some commercially available inactivated vaccines for avian influenza have been used (Nichol and Treanor, 2006; Swayne, 2009). Nonetheless, the global community is probably not well prepared for the next influenza pandemic because viruses have acquired resistance to currently available antiviral drugs. Excessive reliance on vaccines is also inadequate. Vaccines for human and avian influenza have not been accepted worldwide because of their varied protection levels, and traditional vaccines must be updated periodically to account for the antigenic drift and shift of circulating viruses (Lambert and Fauci, 2010). Moreover, the production of a vaccine for a newly emerging strain takes several weeks or months (Lambert and Fauci, 2010). During this time, a pandemic virus can spread globally. Therefore, novel therapeutic schedules are necessary to explore, develop, and control the dynamic and increasingly complicated ecology of circulating IAVs.

Medicinal plants have been widely used to treat various infectious and non-infectious ailments. Natural plant polysaccharides, which are polymeric carbohydrate molecules composed of long chains of monosaccharide units, have different biological activities, including anti-inflammatory activities, immunological regulation, oxidation resistance, and antiviral activities. Numerous studies have reported the inhibitory effects of plant polysaccharides, such as brown algal polysaccharides, *Auricularia auricula* polysaccharides, *Pinus massoniana* pollen polysaccharides, and *Acanthopanax sciadophylloides* polysaccharides, on the replication of human and animal viruses (Queiroz et al., 2008; Nguyen et al., 2012; Lee et al., 2015; Yu et al., 2017). In our study, we focused on *Aloe vera*, which is a perennial Liliaceae evergreen herbaceous plant widely grown in the tropical and subtropical regions. *A. vera* gel contains a large amount of bioactive ingredients, such as vitamins, amino acids, trace elements, polysaccharides, and anthraquinones; as such, it has antibacterial, anti-inflammatory, antioxidant, wound healing-promoting, and immunity-enhancing functions (Langmead et al., 2004; Yagi and Byung, 2015; Kumar and Tiku, 2016). *A. vera* L. can function as nutritional support for patients who are infected with human immunodeficiency virus in clinical trials and can simultaneously affect the viral capability of replication (Radha and Laxmipriya, 2015). *A. vera* also shows antiviral activity against herpes simplex virus, and *A. vera* gel extracts are conducive to the treatment of genital herpes in males (Syed et al., 1996; Zandi et al., 2007). Moreover, *A. vera* can be added to drugs for the treatment of high risk Human papillomavirus infection (Iljazovic et al., 2006). *Aloe* polysaccharides (APS) are active ingredients with a high content in *A. vera* gel. Acemannan polysaccharide is a representative acetylated mannan extracted from *A. vera* gel and has been approved by the US FDA for the treatment of AIDS in humans (Kahlon et al., 1991a,b). Gauntt et al. (2000) also reported that *Aloe* mannan can increase the titers of specific antibodies in mice infected with Coxsackievirus B3, thereby inducing antiviral effects. Therefore, we speculate that APS may have great potential to inhibit influenza virus infection.

This study aimed to explore the inhibitory effect and mechanism of APS on influenza virus infection. We extracted and purified APS from *A. vera* leaves, then we analyzed the monosaccharide composition. We next examined for the first time the anti-influenza activities of APS in a cell culture *in vitro*. Transmission electron microscopy (TEM) was further employed to examine the possible mechanism. The anti-influenza effect of APS was also evaluated in a mouse model. The performance of APS indicated its potential for the development of novel anti-influenza drugs.

## Materials and Methods

### Ethics Statement

The animal procedures were approved by the Animal Care and Use Committee of Shandong Agricultural University (Permit number: 20010510), and performed according to the “Guidelines for Experimental Animals” of the Ministry of Science and Technology (Beijing, China).

### Virus, Cells, and Polysaccharide

Influenza A/Puerto Rico/8/34 (PR8, subtype H1N1) was kept in our laboratory and titrated in MDCK cells by determining TCID_50_. MDCK cells were maintained in Dulbecco’s modified Eagle’s medium (Gibco) containing 10% fetal bovine serum (Gibco) in 5% CO_2_ at 37°C. APS was extracted from *A. vera* leaf through water extraction and ethanol precipitation, and the key steps refer to the method of our previous study (Sun et al., 2011). Briefly, dried *A. vera* leaves and water were mixed at the ratio of 1:10 and extracted at 85°C for 8 h. The extraction liquid was condensed by rotary evaporator. Then high speed centrifugation (12,000 rpm, 20 min) were performed to remove the impurities. Subsequently, ethanol was added into the concentrated solution to precipitate polysaccharides with the final concentration of 75%. After centrifugation and redissolution of polysaccharides, the ethanol precipitation process is repeated three times. Subsequently, the removal of protein was performed Sevag method [chloroform: *n*-butanol = 5:1 (V/V)], and this process was repeated five times. And finally via vacuum drying, the refined APS were obtained.

### Determination of Molecular Weight (Mw) and Monosaccharide Composition

The Mw and monosaccharide composition of APS were determined by Qingdao Sci-tech Innovation Quality Testing Co., Ltd. (Qingdao, China). The Mw was measured by gel permeation chromatography (GPC) method. The monosaccharide composition was measured by Agilent 1200 high performance liquid chromatograph (HPLC, quaternary pump, autosampler, DAD detector, Agilent LC ChemStation). Briefly, APS was hydrolyzed with trifluoroacetic acid and derivatized with 1-phenyl-3-methyl-5-pyrazolone (PMP) and NaOH. Subsequently, HCl was added for neutralization, and monosaccharide extraction was performed thrice with chloroform, then the sample was tested. The monosaccharide standards include 10 kinds of monosaccharides, namely rhamnose, arabinose, galactose, xylose, glucose, mannose, ribosome, fucose, glucuronic acid, and galacturonic acid (Dr. Ehrenstorfer GmbH, Germany). Chromatographic column: Thermo C18 column (4.6 mm × 250 mm, 5.0 μm); Mobile phase: 0.1 mol/L of phosphate buffer solution (pH 7.0):acetonitrile = 82:18 (v/v); The flow rate: 1.0 mL/min; column temperature: 25°C; Sample quantity for 10 μL; wavelength: 245 nm.

### Cytotoxicity Test of APS

MDCK cell monolayers were cultured in 96-well cell culture plates. Different APS concentrations (i.e., 20, 40, 80, 160, 320, and 640 μg/mL) were separately added to the cells. Three repeats were set for each concentration. After 72 h of incubation, the cell viability was determined with MTT assay, and the wavelength for reading was set at 490 nm (Mosmann, 1983).

### Antiviral Activities of APS in MDCK Cells

The extracted APS was diluted to 40, 80, and 160 μg/mL with a maintenance medium (MM). The MDCK cell monolayers grown in 24-well plates were covered with 1.0 mL of MM containing the corresponding APS concentrations and infected with PR8 virus [multiplicity of infection (MOI) = 1]. After 1 h of viral adsorption at 37°C, the supernatants in the wells were replaced with the fresh MM containing the corresponding APS concentrations. Then, the growth dynamics of the virus were detected, and the viral titers of the cell culture supernatant collected every 12 h were determined.

The MDCK cells grown in 24-well plates were inoculated with PR8 virus (MOI = 1) and treated with APS at a final concentration of 40 μg/mL at the following artificially divided viral infection phases to investigate the action phase of APS in the viral life cycle:

#### Before Adsorption (BA)

H1N1 virus was preincubated with APS for 1 h at 4°C and subsequently used for infection. The MDCK cells were infected with the complex of H1N1 virus and polysaccharide for another 1 h at 37°C. After the supernatant was removed, the cells were washed twice and recovered with pure MM.

#### Adsorption (Ad)

MDCK cells were exposed to MM containing the virus and APS for 1 h at 37°C. After the supernatant was removed, the cells were washed twice and recovered with pure MM.

#### After Adsorption (AA)

MDCK cells were infected with H1N1 virus in the absence of APS. After viral adsorption occurred for 1 h at 37°C, the non-adherent viruses were removed. The cells were washed twice and subsequently incubated with MM containing APS.

All of the supernatants in the cell culture wells were collected at each 24 h interval and titrated through TCID_50_ assay. Indirect immunofluorescence assay was performed to identify the virus-infected cells by using the mouse anti-influenza NP monoclonal antibody (Abcam, Cambridge, United Kingdom) at 4 days post-infection. The MDCK cells infected with H1N1 virus in the absence of APS in the entire assay served as the control.

### TEM Assay

The MDCK cell monolayers were infected with PR8 virus suspension (MOI = 1) containing APS (40 μg/mL). The monolayers were scraped at 4 h post-infection and successively fixed in 2.5% glutaraldehyde and 1% osmium tetroxide at 4°C. The cell samples were dehydrated in a graded acetone series prior to infiltration and embedding. Ultrathin (50 nm, LKB-V) longitudinal sections were prepared after the samples were located in the semithin section, stained with uranyl acetate and lead citrate, and examined under a JEOL-1200EX electron microscope (JEOL, Japan). The infected cells without APS treatment serve as the control. Additionally, the PR8 virus suspension was concentrated through sucrose density gradient centrifugation. The isovolumetric virus suspension (0.55 mg/mL) and APS (40 μg/mL) were mixed and incubated for 1 h at 4°C. The mixture was subsequently placed on carbon-coated grids and negatively stained with 0.01 mL of 2% phosphotungstic acid for 1 min. The samples were washed, dried, and examined under a JEOL-1200EX electron microscope. The isovolumetric mixture containing the virus and the STE buffer solution was used as the control.

### Animal Experiment

Eighty female SPF BALB/c mice (7–8 weeks old) were randomly separated into four sterilized isolators, and each isolator contained one group (groups I to IV) comprising 20 mice. The mice were allowed to acclimatize for 3 days before the start of the experiments. All of the mice in groups I, II, and III were infected intranasally with 10^5^ TCID_50_ of the virus. Then, the mice in groups I and II were orally administered with 20 and 40 mg of APS per day, respectively, and the mice in group III were orally treated with isometric PBS. All mice in these three groups were administered continuously for 5 days. The mice in group IV (virus and polysaccharide free) served as the mock control. Groups I, II, III, and IV were called AP (20 mg/day), AP (40 mg/day), PBS, and Mock, respectively. The clinical symptoms and body weight loss of the mice were recorded daily up to 14 days post-infection (dpi). Additionally, another 20 normal mice in each group were placed in new isolators and then infected intranasally with 10^6^ TCID_50_ of the virus (a lethal dose), and the survival rate was monitored up to 7 dpi.

### Detection of Pathological Changes and Viral Loads of the Lungs

Three lung tissues from each group were collected randomly at 3, 5, and 7 dpi and finely ground with a stroke-physiological saline solution at 1:10 (weight:volume). The viral titers in the lung tissues were determined by TCID_50_. At 7 dpi, the pulmonary pathological section was obtained and observed through H&E staining. At the same time, viral localization in lung tissues was performed to observe the distribution of virus-infected cells in the tissue sections through immunofluorescence histochemical staining by using mouse anti-influenza NP monoclonal antibody (Abcam, Cambridge, United Kingdom).

### Statistical Analysis

The data were expressed as mean ± SD, and SPSS 17.0 software was used for statistical evaluation. Duncan’s multiple-range test was used to determine the differences among the groups. Statistical significance was considered at *P* < 0.05.

## Results

### Molecular Weight (Mw) and Monosaccharide Composition of APS

Prior to the experiment, we detected the ingredient composition of APS. The Mw and monosaccharide composition of APS were determined by GPC and HPLC methods, respectively. As shown in **Tables 1, 2**, the Number-average Mw (Mn), Weight-average Mw (Mw), and Z-average Mw (Mz) were 5354, 9198, and 16817 KDa, respectively; the monosaccharide contents of mannose, ribose, rhamnose, glucuronic acid, galacturonic acid, glucose, galactose, xylose, arabinose, and fucose in APS were 5.85, 1.50, 2.20, 5.78, 7.84, 7.57, 52.88, 0, 10.07, and 6.30%, respectively. Usually an antiviral activity of polysaccharide fractions correlates with the molecular mass of the chain (Ghosh et al., 2009). The higher the average Mw (usually ranging from 1 to 500 kDa), the higher is the antiviral activity in many cases, while above 100 kDa, no further increase in activity was observed (Witvrouw and De Clercq, 1997). From the Mw distribution result, approximately 91% of the APS fractions have a molecular weight less than 500 kDa, implying its potential antiviral effects.

**Table 1:** The Mw determination of APS.

Mw (KDa)	Polydispersity (PD):	1.718
	Number-average Mw (Mn):	5354
	Weight-average Mw (Mw) :	9198
	Z-average Mw (Mz) :	16817

Mw distribution	<100, %	76.823
	100∼500, %	14.213
	500∼1000, %	5.624
	1000∼5000, %	1.607
	5000∼10000, %	0.959
	>10000, %	0.774


**Table 2:** The relative content of monosaccharide composition in APS.

Numerical order	Monosaccharide type	Monosaccharide content (%)
1	Mannose	5.85
2	Ribose	1.50
3	Rhamnose	2.20
4	Glucuronic acid	5.78
5	Galacturonic acid	7.84
6	Glucose	7.57
7	Galactose	52.88
8	Xylose	0 (not detected)
9	Arabinose	10.07
10	Fucose	6.30


### APS Inhibits Replication of PR8 Virus *in vitro*

**FIGURE 1:**
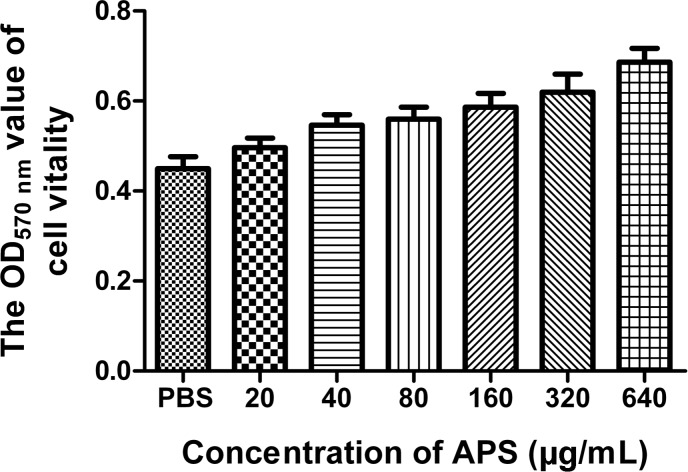
Influence of APS on cell activity. MDCK cells were cultured with the various concentrations of APS (20, 40, 80, 160, 320, and 640 μg/mL) or PBS for 72 h. The cell viability was determined by MTT assay.

**FIGURE 2:**
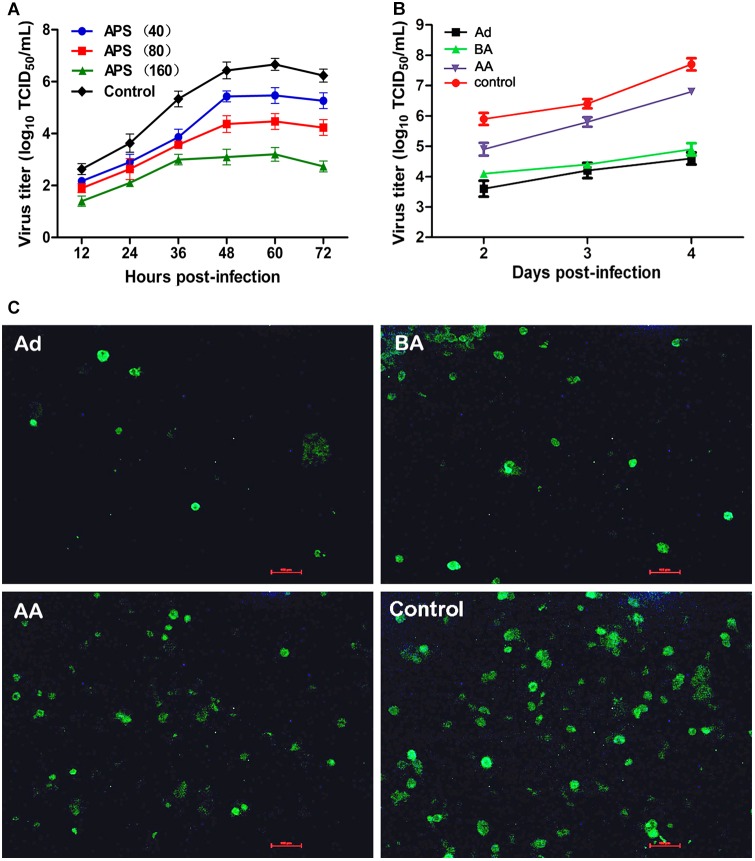
*Aloe* polysaccharides (APS) inhibits influenza virus replication in MDCK cells. **(A)** The MDCK cells were cultured with medium containing different concentrations of APS and then infected with PR8 virus [(MOI) = 1]. The virus titers of the culture supernatant collected each 12 h were determined by TCID_50_. The number in parentheses represent the concentration of drug; **(B)** MDCK cells infected with PR8 virus [(MOI) = 1] were treated with 40 μg/mL of APS at different viral infection phases [before adsorption (BA), adsorption (Ad), and after adsorption (AA)] in the whole infection process. Supernatants were collected at each 24 h interval and titrated by TCID_50_. **(C)** IFA detection of PR8 virus infected cells treated with APS at 4 days after inoculation. PBS treated cell wells served as the control. All values shown are presented as means ± SD from three independent experiments.

We first determined the viral replication kinetics in the MDCK cells treated with different APS concentrations to examine the antiviral characteristics of APS against the H1N1 (PR8) virus. Prior to the experiment, the toxicity of APS to MDCK cells was examined through MTT method. The results showed that APS at concentrations ranging from 20 to 640 μg/mL elicited no significant cytotoxicity on cellular activities. Conversely, the existence of APS promoted the cell growth in a dose-dependent manner (**Figure 1**). In the viral replication kinetics assay, the viral titers in the cell cultures containing 40, 80, and 160 μg/mL of APS were significantly lower than those in the control group from 24 to 72 h post-infection (*P* < 0.05; **Figure 2A**). We observed that the antiviral activity of APS remained in a typical dose-dependent manner.

We chose a minimum effective concentration above (40 μg/mL) to determine the reaction phase of APS in the viral life cycle and to evaluate its antiviral effects at three artificially divided viral infection phases, namely, before adsorption (BA), adsorption (Ad), and after adsorption (AA). We determined that the viral titers of BA, Ad, and AA groups were lower than those of the non-polysaccharide-treated control group in the entire monitoring period. Minimum viral titers were detected in the Ad group (*P* < 0.05; **Figure 2B**), indicating the optimal antiviral effect of APS administered in the viral adsorption phase. The viral titers in the BA group also showed an obvious suppressive effect compared with that of the control group, indicating that the preincubation of the virus and polysaccharide could interfere with viral replication.

Similarly, the intensity and density of antigen-reactive immunofluorescence in the Ad and BA groups were significantly lower than those in the AA and control groups at 4 dpi (*P* < 0.05; **Figure 2C**). These results showed that APS could remarkably inhibit replication of PR8 virus *in vitro*, and the inhibitory effect was optimal at the viral adsorption phase.

### Interaction Between APS and PR8 Virus

**FIGURE 3:**
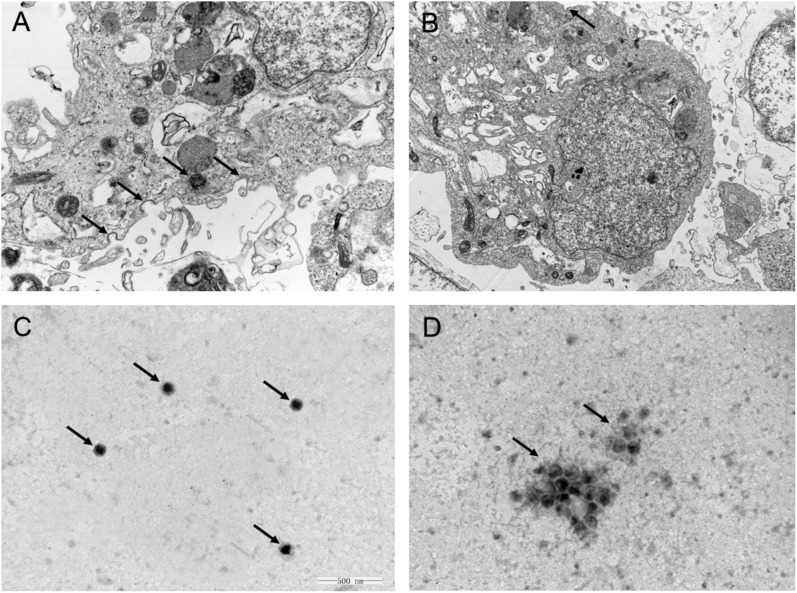
*Aloe* polysaccharides interferes with viral adsorption to MDCK cells. MDCK monolayers were infected with MOI = 1 of PR8 virus in the absence **(A)** or presence **(B)** of APS treatment (40 μg/mL). At 4 h post-infection, the cells were centrifuged and fixed to prepare ultrathin sections, and the virions in cells were imaged via TEM (30000×). The virions on the cell membrane surface and in the endosomes were denoted by black arrowheads. Te purified PR8 virus **(C)** and the mixture of virus and APS **(D)** were imaged via TEM after negative staining (60000×). The virions were denoted by black arrowheads.

One feasible way for the reported plant polysaccharides to inhibit viral infection is to interfere with viral adsorption (Harden et al., 2009; Yu et al., 2017). Considering the previously presented results, we determined whether APS inhibited H1N1 infection in this manner. The viral adsorption capability of the MDCK cells treated with APS was assessed through TEM tomography. **Figure 3A** shows that several virus particles in the membrane fusion phase were observed on the cell membrane surface of the infected cells without APS treatment. By contrast, few H1N1 virus particles were observed on the membrane surface when treated with APS (**Figure 3B**). Moreover, some visible virions in the virus-infected cells were distributed densely in the endosomes (**Figure 3A**), whereas few virions were observed in the endosomes of the APS-treated cells (**Figure 3B**). This phenomenon indicated that APS reduced viral adsorption to and infection of the host cells.

Considering the previous findings (Song et al., 2013; Yang et al., 2015), we speculated that APS might act directly on the virus. As such, we conducted TEM to investigate the interaction between APS and virus particles. The scattered virus particles in the purified virus sample appeared as spheroidal structures with an inner, centrally located electron-dense core, and the average size of the virus particles was approximately 110 nm (**Figure 3C**). By contrast, the virus particles treated with APS accumulated into clusters, and the virions exhibited irregular shapes and comprised low or no electron density cores and scattered fragments around the cluster (**Figure 3D**). These results revealed a direct effect of APS on the PR8 virus particles.

### APS Reduces the Pathogenicity of the PR8 Virus

**FIGURE 4:**
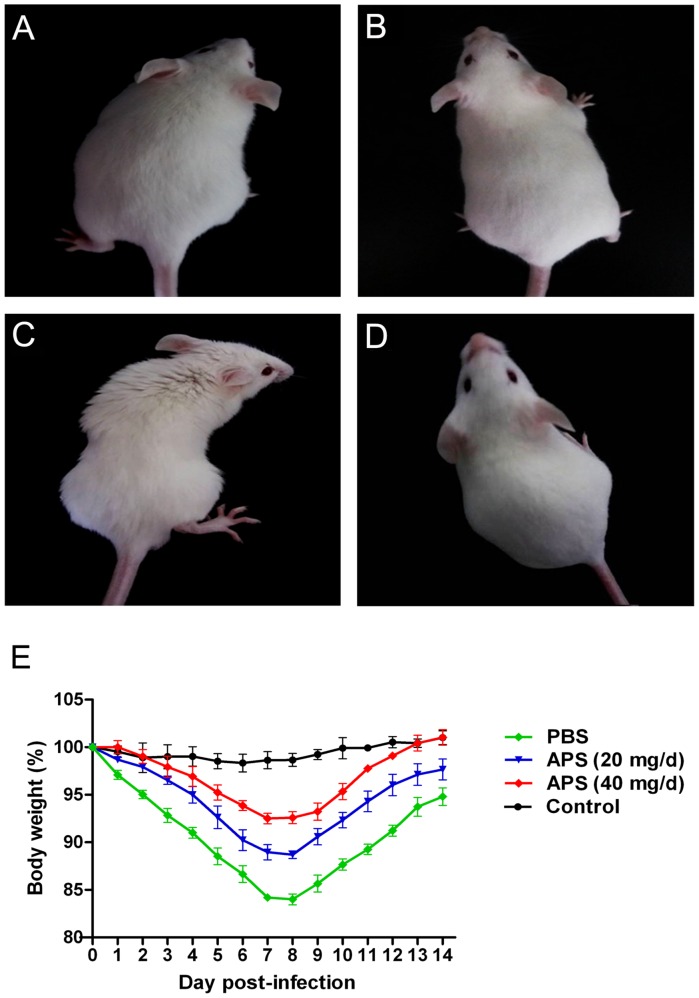
*Aloe* polysaccharides reduces the clinical symptoms of infected mice. All 60 mice in groups I **(A)**, II **(B)**, and III **(C)** were infected intranasally with the 10^5^ TCID_50_ of virus. Then the mice in groups I and II were orally administrated with 20 and 40 mg of APS per day, respectively, and group III were orally administrated isometric PBS. Another 20 virus-free mice in groups IV **(D)** served as the normal control. The typical clinical symptoms were recorded at 7 dpi **(A–D)**, and the body weight loss **(E)** of animals were recorded every day up to 14 dpi.

We administered APS after the mice were challenged with the PR8 virus to investigate whether APS had an inhibitory activity against PR8 virus infection *in vivo*. The clinical signs and pathological changes were monitored. The observation of the clinical symptoms showed that the mice infected with PR8 virus (10^5^ TCID_50_) were listless and had messy, lackluster, and bristled fur (**Figure 4C**). They also exhibited mental depression and poor appetite by knocking on the cage and checking the amount of food left. However, APS administration significantly ameliorated the symptoms of the infected mice. After the treatment with 20 mg/day APS, the infected mice showed slightly messy fur and mild depression (**Figure 4A**). By contrast, the mice treated with 40 mg/day APS exhibited smooth and lustrous fur and normal behavior (**Figure 4B**), which was similar to that of the control group (**Figure 4D**). Consistent with the clinical symptoms, the body weight of the infected mice was significantly low. The loss of body weight of the mice administered with APS was significantly alleviated, and the effect of 40 mg/day APS was better than that of 20 mg/day APS (*P* < 0.05; **Figure 4E**).

**FIGURE 5:**
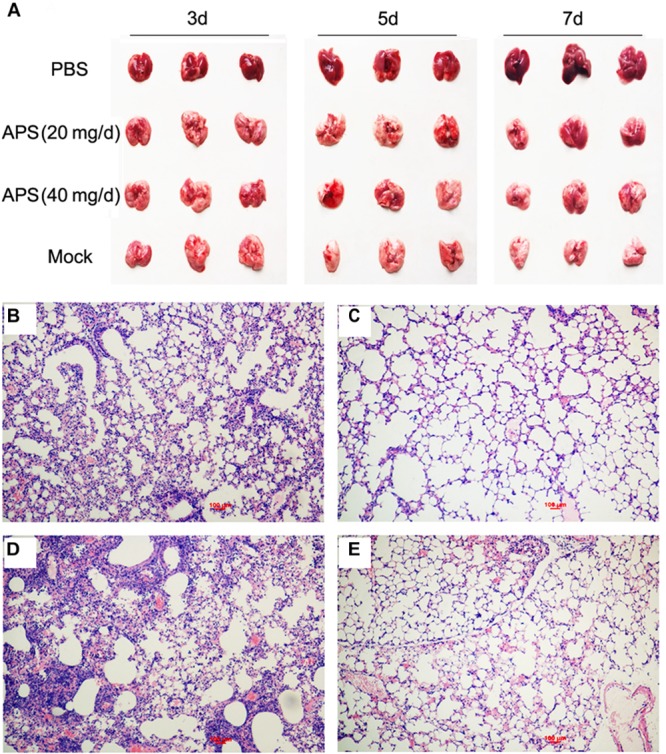
*Aloe* polysaccharides reduces the pathological change of infected lungs. All mice in groups I, II, and III were infected intranasally with the 10^5^ TCID_50_ of virus. Then the mice in groups I and II were orally administrated with 20 and 40 mg of APS per day, respectively, and group III were orally administrated isometric PBS. The virus-free mice in groups IV served as the normal control. At 7 dpi, the lungs in each group were collected, and the pulmonary lesions **(A)** and pathological changes (**B**, group I; **C,** group II; **D**, group III; **E**, group IV) were compared through subjective observation and H&E staining, respectively. Scale bar, 100 μm.

Most animals and even humans who died of influenza virus infection developed acute respiratory distress syndrome, which is the most severe form of acute lung injury (Tumpey et al., 2005), and the pathogenicity is characterized by inflammatory cell accumulation, edema formation, and marked cytokine increase. A pathologic examination was performed postmortem to examine the pathological changes in the mice treated or untreated with APS against avian influenza infection. PR8-virus-infected lungs had severe lesions with extensive consolidation in all of the lobes (**Figure 5A**). The APS-treated mice did not significantly develop acute lung injury after they were infected with virus (**Figure 5A**). Furthermore, the infected lungs from different groups were excised for histopathological evaluation at 7 dpi. The microscopic lesions of the virus-infected lungs were severe peribronchiolitis and bronchopneumonia, which were characterized by edema and diffuse infiltration of inflammatory cells in the alveolar lumen and bronchioles (**Figure 5D**). However, APS treatment significantly alleviated the pulmonary histopathologic symptoms (**Figures 5B,C**). The mice treated with 40 mg/day APS had mild lesions in the lungs, which even resembled the lungs of the control group (**Figure 5E**). These results indicated that APS largely alleviated the clinical symptoms and pulmonary lesions induced by influenza virus infection.

### APS Reduces the Viral Loads and Mortality of Virus-Infected Mice

**FIGURE 6:**
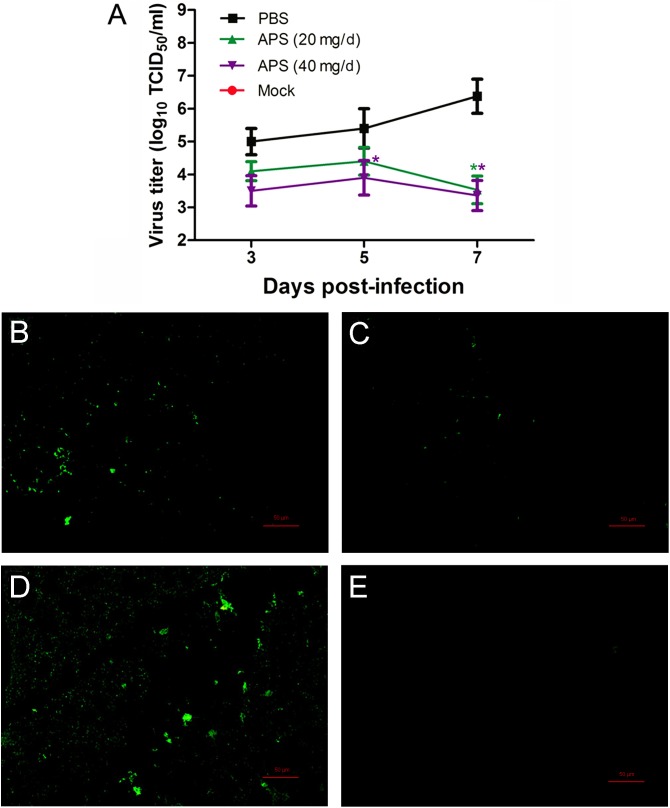
*Aloe* polysaccharides reduces viral loads in virus-infected mice. All mice in groups I, II, and III were infected intranasally with the 10^5^ TCID_50_ of virus. Then the mice in groups I and II were orally administrated with 20 and 40 mg of APS per day, respectively, and group III were orally administrated isometric PBS. The virus-free mice in groups IV served as the normal control. The viral titers **(A)** in lung tissues were determined by TCID50. The viral localization in lung tissues sections was also performed through immunofluorescence histochemical staining using the mouse anti-influenza NP monoclonal antibody (200×; **B**, group I; **C**, group II; **D**, group III; **E**, group IV). Scale bar, 50 μm. An asterisk indicates that the value of the corresponding group was significantly different from that of the group III (*P* < 0.05).

**FIGURE 7:**
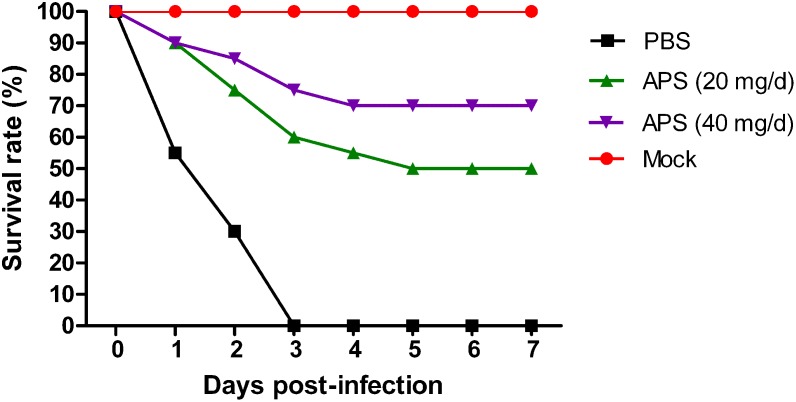
*Aloe* polysaccharides reduces mortality in virus-infected mice. Twenty mice in groups I, II, and III were infected intranasally with the 10^6^ TCID_50_ of virus. Then the mice in groups I and II were orally administrated with 20 and 40 mg of APS per day, respectively, and group III were orally administrated isometric PBS. The virus-free mice in groups IV served as the normal control. The survival rate was monitored up to 7 dpi.

We determined the degree of virus propagation in PR8-infected mice with or without APS treatment to investigate the practical virustatic effects of APS *in vivo* further. The influenza virus mainly exists in the lungs of infected animals and mediates the damage of the airway, alveolar epithelium, and alveolar endothelium (Herold et al., 2015). Thus, lung tissues from different groups were collected, and the viral titers were analyzed at 3, 5, and 7 dpi. The viral titers in the lung tissues of the 40 mg/day APS treated groups significantly decreased from 5 to 7 dpi compared with that in the control group, and those of the 20 mg/day APS treated groups by 7 dpi (*P* < 0.05; **Figure 6A**). The viral colonization in the lung tissues fixed at 7 dpi was also visualized through immunofluorescence histochemical staining by using an anti-influenza NP monoclonal antibody and fluorescent secondary antibodies. We observed intense viral antigen staining in the section of the lung tissue infected with PR8 virus (**Figure 6D**). The staining densities in the lung tissue sections of the APS-treated groups (**Figures 6B,C**) were obviously lower than those of the PBS group (**Figure 6D**), and few positively stained lung cells were observed in the section administered with 40 mg/day APS (**Figure 6C**). No viral signals were detected in the virus-free control group (**Figure 6E**). Moreover, the mortality of the mice treated with 40 mg/day APS declined by 70% compared with the mice without APS treatment after infection with a lethal dose of 10^6^ TCID_50_ (**Figure 7**). These results indicated that mice orally administrated with APS after virus infection could be protected from the lethal infection of the PR8 virus.

## Discussion

Seasonal influenza is a long-term threat to human health, and it causes significant morbidity and mortality every year. Exploring new ways to prevent and treat IAV infection is crucial to control influenza outbreak. In the current study, we examined the ingredient composition of APS and determined that APS exerted a significant antiviral activity to an H1N1 subtype influenza virus *in vitro* and *in vivo*. The TEM assay demonstrated that APS interacted with influenza virions, thereby preventing the attachment of the virus to the host cells. In the *in vivo* experiment, APS obviously reduced viral shedding and viral loads in mouse lungs and ameliorated the clinical symptoms and mortality of influenza virus-infected mice.

Influenza A virus is an ineradicable contagious disease causing seasonal epidemic and sporadic pandemic outbreaks that pose significant morbidity and mortality for humans and animals. IAV can be transmitted through aerosols or respiratory droplets, resulting in respiratory infections transmitted via air droplets and further inducing pneumonia (La Gruta et al., 2007; Bouvier and Palese, 2008; Kuiken et al., 2012). Herein, we determined that APS significantly induced an anti-influenza virus effect *in vitro* and an evident therapeutic effect on virus-infected mice. A dose-dependent antiviral effect was observed in cell assay. In particular, the effects were most pronounced in the early stages of infection. Given this characteristic, nasal administration may also be an effective method. Besides, APS administration alleviated pulmonary congestion, edema, and inflammatory cell infiltration and decreased the viral titers in lung tissues. These phenomena verify our speculations about the anti-influenza activity of APS. Currently, existing anti-flu drugs, such as oseltamivir and zanamivir, usually work on a particular protein of the influenza virus and can therefore easily induce drug resistance (Hata et al., 2008; Jefferson et al., 2014). However, this drawback can be effectively avoided by plant polysaccharides, which are complex macromolecules.

Acemannan and polymannose extracted from *Aloe* plants have antiviral activities as described in the Section “Introduction,” and some possible mechanisms have been investigated. Saoo et al. (1996) reported the antiviral activity of *A. barbadensis* Miller against human cytomegalovirus (HCMV) and showed that the major mechanism of *Aloe* extracts in inhibiting HCMV infection involves the interference of DNA synthesis. The antiviral activity of *Aloe* emodin against IAV has also been reported (Li et al., 2014). Recently, a study shows that emodin can inhibit IAV replication and influenza viral pneumonia by activating Nrf2 signaling and inhibiting IAV-induced activation of TLR4, p38/JNK MAPK, and NF-κB pathways (Dai et al., 2017). However, the antiviral mechanism of APS is poorly understood.

To our knowledge, the antiviral mechanism of plant-origin polysaccharides is a complex process. Studies have shown three possible antiviral mechanisms of plant polysaccharides. First, polysaccharides directly interfere with viral infection in host cells (Ghosh et al., 2009; Yu et al., 2017). Second, polysaccharides induce the expression of relevant host antiviral proteins, i.e., intracellular signaling pathways (Rechter et al., 2006; Ghosh et al., 2009). Third, polysaccharides have immunoregulatory activity, for instance, the ability of APS to improve host immunity has been explored, and Lee et al. (2001) proposed that acemannan, a major carbohydrate fraction of *A. vera* gel, can promote the differentiation of immature DCs and exert immunomodulatory activity (Sun et al., 2011). Here, our *in vitro* experiments confirmed that the key time of APS action is the viral adsorption period because most virions have not yet entered host cells at this time. Therefore, APS may interfere with influenza virus adsorption by an unknown mechanism. On the basis of this assumption, we further determined the direct antiviral mechanism of APS by investigating the interaction between APS and influenza virions via the TEM assay. The results showed that APS directly affected the morphological characteristics and distribution of influenza particles. Notably, we observed a peculiar phenomenon in which the IAV particles clustered and exhibited irregular shapes and scattered fragments after these particles interacted with APS. This phenomenon was similar to that observed in our previous study of the interaction between Taishan *P. massoniana* pollen polysaccharide and avian leukemia virus (Yu et al., 2017). Therefore, the direct interaction between APSs and IAVs is a likely mechanism of inhibiting viral infection.

## Conclusion

Polysaccharides extracted from *A. vera*, a common medicinal plant, elicited significant anti-influenza virus effects *in vivo* and *in vitro*. APS could directly interact with PR8 (H1N1) influenza virus particles to prevent virus particle adsorption. Our previous studies have also shown that APS can increase the activity of the immune system, which is another important mechanism of antiviral infection. In future studies, more virus strains and hosts should be used for further validation, and signaling pathways associated with APS interaction will be investigated to explain their unknown anti-influenza mechanism. This study provided a critical theoretical basis for the development of APS as a novel anti-influenza drug.

## Author Contributions

KW, JZ, and ZS designed the research. ZS, CY, WW, GY, TZ, and LZ performed the research. ZS, CY, KW, and JZ analyzed the data and wrote the paper.

## Conflict of Interest Statement

The authors declare that the research was conducted in the absence of any commercial or financial relationships that could be construed as a potential conflict of interest.

## References

[B1] Bouvier N. M. Palese P. (2008). The biology of influenza viruses. *Vaccine* 26 D49–D53. 19230160 10.1016/j.vaccine.2008.07.039 PMC3074182

[B2] Dai J.-P. Wang Q.-W. Su Y. Gu L.-M. Zhao Y. Chen X.-X. (2017). Emodin Inhibition of Influenza A Virus Replication and Influenza Viral Pneumonia via the Nrf2, TLR4, p38/JNK and NF-kappaB Pathways. *Molecules* 22:E1754. 10.3390/molecules22101754 29057806 PMC6151665

[B3] Gauntt C. Wood H. McDaniel H. McAnalley B. (2000). Aloe polymannose enhances anti-coxsackievirus antibody titres in mice. *Phytother. Res.* 14 261–266. 10.1002/1099-1573(200006)14:4<261::AID-PTR579>3.0.CO;2-A 10861969

[B4] Ghosh T. Chattopadhyay K. Marschall M. Karmakar P. Mandal P. Ray B. (2009). Focus on antivirally active sulfated polysaccharides: from structure–activity analysis to clinical evaluation. *Glycobiology* 19 2–15. 10.1093/glycob/cwn092 18815291

[B5] Harden E. A. Falshaw R. Carnachan S. M. Kern E. R. Prichard M. N. (2009). Virucidal activity of polysaccharide extracts from four algal species against herpes simplex virus. *Antiviral Res.* 83 282–289. 10.1016/j.antiviral.2009.06.007 19576248 PMC2730659

[B6] Hata K. Koseki K. Yamaguchi K. Moriya S. Suzuki Y. Yingsakmongkon S. (2008). Limited inhibitory effects of oseltamivir and zanamivir on human sialidases. *Antimicrob. Agents Chemother.* 52 3484–3491. 10.1128/AAC.00344-08 18694948 PMC2565904

[B7] Herold S. Becker C. Ridge K. M. Budinger G. S. (2015). Influenza virus-induced lung injury: pathogenesis and implications for treatment. *Eur. Respir. J.* 45 1463–1478. 10.1183/09031936.00186214 25792631

[B8] Hibino A. Kondo H. Masaki H. Tanabe Y. Sato I. Takemae N. (2017). Community-and hospital-acquired infections with oseltamivir-and peramivir-resistant influenza A (H1N1) pdm09 viruses during the 2015–2016 season in Japan. *Virus Genes* 53 89–94. 10.1007/s11262-016-1396-9 27714496 PMC5306182

[B9] Iljazovic E. Zulcic-Nakic V. Latifagic A. Sahimpasic A. Omeragic F. Avdic S. (2006). 245 ORAL Efficacy in treatment of cervical HRHPV infection by combination of interferon, Aloe vera and propolis gel associated with different cervical lesion. *Eur. J. Surg. Oncol.* 32 S73–S139. 10.1016/S0748-7983(06)70680-1

[B10] Jefferson T. Jones M. A. Doshi P. Del Mar C. B. Hama R. Thompson M. (2014). Neuraminidase inhibitors for preventing and treating influenza in healthy adults and children. *Sao Paulo Med. J.* 132 256–257. 10.1590/1516-3180.20141324T2 25055075 PMC10496739

[B11] Kahlon J. Kemp M. Carpenter R. McAnalley B. McDaniel H. Shannon W. (1991a). Inhibition of AIDS virus replication by acemannan in vitro. *Mol. Biother.* 3 127–135. 1768365

[B12] Kahlon J. Kemp M. Yawei N. Carpenter R. Shannon W. McAnalley B. (1991b). In vitro evaluation of the synergistic antiviral effects of acemannan in combination with azidothymidine and acyclovir. *Mol. Biother.* 3 214–223. 1662957

[B13] Kuiken T. Riteau B. Fouchier R. Rimmelzwaan G. (2012). Pathogenesis of influenza virus infections: the good, the bad and the ugly. *Curr. Opin. Virol.* 2 276–286. 10.1016/j.coviro.2012.02.013 22709515

[B14] Kumar S. Tiku A. B. (2016). Immunomodulatory potential of acemannan (polysaccharide from Aloe vera) against radiation induced mortality in Swiss albino mice. *Food Agric. Immunol.* 27 72–86. 10.1080/09540105.2015.1079594

[B15] La Gruta N. L. Kedzierska K. Stambas J. Doherty P. C. (2007). A question of self-preservation: immunopathology in influenza virus infection. *Immunol. Cell Biol.* 85 85–92. 10.1038/sj.icb.7100026 17213831

[B16] Lambert L. C. Fauci A. S. (2010). Influenza vaccines for the future. *N. Engl. J. Med.* 363 2036–2044. 10.1056/NEJMra1002842 21083388

[B17] Langmead L. Makins R. Rampton D. (2004). Anti-inflammatory effects of aloe vera gel in human colorectal mucosa in vitro. *Aliment. Pharmacol. Ther.* 19 521–527. 10.1111/j.1365-2036.2004.01874.x 14987320

[B18] Lee J.-B. Tanikawa T. Hayashi K. Asagi M. Kasahara Y. Hayashi T. (2015). Characterization and biological effects of two polysaccharides isolated from *Acanthopanax sciadophylloides*. *Carbohydr. Polym.* 116 159–166. 10.1016/j.carbpol.2014.04.013 25458285

[B19] Lee J. K. Lee M. K. Yun Y.-P. Kim Y. Kim J. S. Kim Y. S. (2001). Acemannan purified from Aloe vera induces phenotypic and functional maturation of immature dendritic cells. *Int. Immunopharmacol.* 1 1275–1284. 10.1016/S1567-5769(01)00052-2 11460308

[B20] Leung K. Lipsitch M. Yuen K. Y. Wu J. T. (2017). Monitoring the fitness of antiviral-resistant influenza strains during an epidemic: a mathematical modelling study. *Lancet Infect. Dis.* 17 339–347. 10.1016/S1473-3099(16)30465-0 27914853 PMC5470942

[B21] Li S.-W. Yang T.-C. Lai C.-C. Huang S.-H. Liao J.-M. Wan L. (2014). Antiviral activity of aloe-emodin against influenza A virus via galectin-3 up-regulation. *Eur. J. Pharmacol.* 738 125–132. 10.1016/j.ejphar.2014.05.028 24877694

[B22] Liu S. S. Jiao X. Y. Wang S. Su W. Z. Jiang L. Z. Zhang X. (2017). Susceptibility of influenza A (H1N1)/pdm2009, seasonal A (H3N2) and B viruses to Oseltamivir in Guangdong, China between 2009 and 2014. *Sci. Rep.* 7:8488. 10.1038/s41598-017-08282-6 28814737 PMC5559489

[B23] Ly S. Horwood P. Chan M. Rith S. Sorn S. Oeung K. (2017). Seroprevalence and transmission of human influenza A (H5N1) virus before and after virus reassortment, Cambodia, 2006–2014. *Emerg. Infect. Dis.* 23:300. 10.3201/eid2302.161232 28098551 PMC5324818

[B24] Mosmann T. (1983). Rapid colorimetric assay for cellular growth and survival: application to proliferation and cytotoxicity assays. *J. Immunol. Methods* 65 55–63. 10.1016/0022-1759(83)90303-4 6606682

[B25] Nguyen T. L. Chen J. Hu Y. Wang D. Fan Y. Wang J. (2012). In vitro antiviral activity of sulfated *Auricularia auricula* polysaccharides. *Carbohydr. Polym.* 90 1254–1258. 10.1016/j.carbpol.2012.06.060 22939338

[B26] Nichol K. L. Treanor J. J. (2006). Vaccines for seasonal and pandemic influenza. *J. Infect. Dis.* 194(Suppl. 2), S111–S118. 10.1086/507544 17163383

[B27] Pan M. Gao R. Lv Q. Huang S. Zhou Z. Yang L. (2016). Human infection with a novel, highly pathogenic avian influenza A (H5N6) virus: virological and clinical findings. *J. Infect.* 72 52–59. 10.1016/j.jinf.2015.06.009 26143617

[B28] Queiroz K. Medeiros V. Queiroz L. Abreu L. Rocha H. Ferreira C. (2008). Inhibition of reverse transcriptase activity of HIV by polysaccharides of brown algae. *Biomed. Pharmacother.* 62 303–307. 10.1016/j.biopha.2008.03.006 18455359

[B29] Radha M. H. Laxmipriya N. P. (2015). Evaluation of biological properties and clinical effectiveness of Aloe vera: a systematic review. *J. Tradit. Complement. Med.* 5 21–26. 10.1016/j.jtcme.2014.10.006 26151005 PMC4488101

[B30] Rechter S. König T. Auerochs S. Thulke S. Walter H. Dörnenburg H. (2006). Antiviral activity of Arthrospira-derived spirulan-like substances. *Antiviral Res.* 72 197–206. 10.1016/j.antiviral.2006.06.004 16884788

[B31] Saoo K. Miki H. Ohmori M. Winters W. (1996). Antiviral activity of aloe extracts against cytomegalovirus. *Phytother. Res.* 10 348–350. 10.1002/(SICI)1099-1573(199606)10:4<348::AID-PTR836>3.0.CO;2-2

[B32] Song X. Yin Z. Li L. Cheng A. Jia R. Xu J. (2013). Antiviral activity of sulfated *Chuanminshen violaceum* polysaccharide against duck enteritis virus in vitro. *Antiviral Res.* 98 344–351. 10.1016/j.antiviral.2013.03.012 23523763

[B33] Sun Z. Wei K. Yan Z. Zhu X. Wang X. Wang H. (2011). Effect of immunological enhancement of aloe polysaccharide on chickens immunized with *Bordetella avium* inactivated vaccine. *Carbohydr. Polym.* 86 684–690. 10.1016/j.carbpol.2011.05.012

[B34] Swayne D. E. (2009). Avian influenza vaccines and therapies for poultry. *Comp. Immunol. Microbiol. Infect. Dis.* 32 351–363. 10.1016/j.cimid.2008.01.006 18442853

[B35] Syed T. A. Cheema K. M. Ahmad S. A. Holt A. H. Jr. (1996). Aloe vera extract 0.5% in hydrophilic cream versus Aloe vera gel for the management of genital herpes in males. A placebo-controlled, double-blind, comparative study. *J. Eur. Acad. Dermatol. Venereol.* 7 294–295.

[B36] Taubenberger J. K. Morens D. M. (2017). H5Nx panzootic Bird Flu—influenza’s newest worldwide evolutionary tour. *Emerg. Infect. Dis.* 23:340 10.3201/eid2302.161963

[B37] Tumpey T. M. Basler C. F. Aguilar P. V. Zeng H. Solórzano A. Swayne D. E. (2005). Characterization of the reconstructed 1918 Spanish influenza pandemic virus. *Science* 310 77–80. 10.1126/science.1119392 16210530

[B38] Witvrouw M. De Clercq E. (1997). Sulfated polysaccharides extracted from sea algae as potential antiviral drugs. *Gen. Pharmacol.* 29 497–511. 10.1016/S0306-3623(96)00563-0 9352294

[B39] Yagi A. Byung P. Y. (2015). Immune modulation of Aloe vera: acemannan and gut microbiota modulator. *J. Gastroenterol. Hepatol. Res.* 4 1707–1721. 10.17554/j.issn.2224-3992.2015.04.525

[B40] Yang S. Wei K. Jia F. Zhao X. Cui G. Guo F. (2015). Characterization and biological activity of Taishan *Pinus* massoniana pollen polysaccharide *in vitro. PLoS One* 10:e0115638. 10.1371/journal.pone.0115638 25782009 PMC4363904

[B41] Ylipalosaari P. Ala-Kokko T. I. Laurila J. Ahvenjärvi L. Syrjälä H. (2017). ICU-treated influenza A (H1N1) pdm09 infections more severe post pandemic than during 2009 pandemic: a retrospective analysis. *BMC Infect. Dis.* 17:728. 10.1186/s12879-017-2829-3 29162037 PMC5697104

[B42] Yu C. Wei K. Liu L. Yang S. Hu L. Zhao P. (2017). Taishan *Pinus* massoniana pollen polysaccharide inhibits subgroup J avian leucosis virus infection by directly blocking virus infection and improving immunity. *Sci. Rep.* 7:44353. 10.1038/srep44353 28287165 PMC5347021

[B43] Zandi K. Zadeh M. A. Sartavi K. Rastian Z. (2007). Antiviral activity of *Aloe* vera against herpes simplex virus type 2: an *in vitro* study. *Afr. J. Biotechnol.* 6.

